# Classification of the Adenylation and Acyl-Transferase Activity of NRPS and PKS Systems Using Ensembles of Substrate Specific Hidden Markov Models

**DOI:** 10.1371/journal.pone.0062136

**Published:** 2013-04-18

**Authors:** Barzan I. Khayatt, Lex Overmars, Roland J. Siezen, Christof Francke

**Affiliations:** 1 Center for Molecular and Biomolecular Informatics, Nijmegen Center for Molecular Life Sciences, Radboud University Nijmegen Medical Centre, Nijmegen, The Netherlands; 2 Netherlands Bioinformatics Center, Nijmegen, The Netherlands; 3 Kluyver Center for Genomics of Industrial Fermentation, Delft, The Netherlands; 4 TI Food and Nutrition, Wageningen, The Netherlands; The University of Hong Kong, China

## Abstract

There is a growing interest in the Non-ribosomal peptide synthetases (NRPSs) and polyketide synthases (PKSs) of microbes, fungi and plants because they can produce bioactive peptides such as antibiotics. The ability to identify the substrate specificity of the enzyme's adenylation (A) and acyl-transferase (AT) domains is essential to rationally deduce or engineer new products. We here report on a Hidden Markov Model (HMM)-based ensemble method to predict the substrate specificity at high quality. We collected a new reference set of experimentally validated sequences. An initial classification based on alignment and Neighbor Joining was performed in line with most of the previously published prediction methods. We then created and tested single substrate specific HMMs and found that their use improved the correct identification significantly for A as well as for AT domains. A major advantage of the use of HMMs is that it abolishes the dependency on multiple sequence alignment and residue selection that is hampering the alignment-based clustering methods. Using our models we obtained a high prediction quality for the substrate specificity of the A domains similar to two recently published tools that make use of HMMs or Support Vector Machines (NRPSsp and NRPS predictor2, respectively). Moreover, replacement of the single substrate specific HMMs by ensembles of models caused a clear increase in prediction quality. We argue that the superiority of the ensemble over the single model is caused by the way substrate specificity evolves for the studied systems. It is likely that this also holds true for other protein domains. The ensemble predictor has been implemented in a simple web-based tool that is available at http://www.cmbi.ru.nl/NRPS-PKS-substrate-predictor/.

## Introduction

In recent years the Non-Ribosomal Peptide Synthetases (NRPSs) and PolyKetide Synthases (PKSs) have gained considerable interest as they can produce polypeptide- and polyketide-based secondary metabolites that exhibit important pharmaceutical and biological activities (see e.g. [1]–[7]). The Synth(et)ases can be found in a wide variety of bacteria, fungi and plants, and produce secondary metabolites that range from antibiotics to kill competitors (like e.g. penicillin and erythromycin), to surfactants to thrive in a biofilm environment (like e.g. surfactin) (for reviews see [8]–[12]). NRPSs and PKSs are large multi-module/domain proteins (protein-systems). The simplest NRPS module consists of at least three core domains: an adenylation domain (A) that selects, activates and loads the substrate (i.e. proteinogenic and non-proteinogenic amino acids); a thiolation domain (T) -which is also known as the peptidyl carrier protein- that covalently attaches the substrate to the synthetase; and finally a condensation domain (C) that catalyzes peptide bond formation. The three core domains of the simplest PKS are: an acyl-transferase domain (AT) that recognizes and loads small carboxylic acid building blocks such as provided by malonyl-CoA or methylmalonyl-CoA; an acyl-carrier protein (ACP) domain that resembles the T domain of NRPSs and retains the building blocks; and a keto-synthase domain (KS) that builds the polyketide chain via condensation. NRPSs and PKSs finally have a fourth domain, the thio-esterase domain (TE) that releases the assembled polypeptide and polyketide chains from the synth(et)ase. The core domains are organized in functional modules and multiple modules make up a kind of assembly-line to construct linear, cyclic or branched secondary metabolites (for a detailed description of the mechanism we refer to the excellent reviews by [9], [13]–[20]). In various cases other enzymes act on the created polypeptide and polyketide chains to tailor the final product (e.g. [21], [22]). These other enzymes are usually associated to the synth(et)ase complex and their genes are often organized in the same gene clusters [23].

The structure and activity of the natural products produced by NRPSs and PKSs are determined by the specific substrates that are bound by the A and AT domains, respectively. Co-crystallization of the malonyl-CoA-specific acyl-transferase [PDB∶1MLA] from *Escherichia coli* fatty acid synthase (FabD) and its substrate, enabled the identification of 13 active site residues in the AT domain [24]. These residues were later proposed, together with 10 adjacent residues, as the substrate specificity-conferring residues by [25]. Similarly, the crystal structure of the phenylalanine-specific A domain [PDB∶1AMU] of gramicidin synthetase A (GrsA) [26] facilitated the identification of 10 residues that line the active site pocket of the A domain, and later these were proposed as a sort of substrate specificity conferring code for the A domain [27], [28].

Most substrate specificity prediction tools that have been developed are based on the A and AT active site residues. The tools include: NRPS–PKS [29], PKS/NRPS Analysis [30], PKSDB [25], NPsearcher [31] and SBSPKS [32]. Other prediction methods have also focused on the active site, albeit that there was more variation in the number of residues that was taken into account. For instance, the NRPS predictor tool that was developed by [33], and that was later implemented in the application CLUSEAN [34], based its prediction on 34 residues in, and close to, the active site of the A domain. The accuracy of various approaches that were available up to 2010 was analyzed by [32]. The authors concluded that the NRPS–PKS interface of their own SBSPKS tool could efficiently predict the substrate linked to malonate- and methylmalonate-specific AT domains with high specificity and sensitivity, and that the results were comparable to those reported by Minowa et al. [35] for substrates that are less common.

The classification and selection procedures described above rely on multiple sequence alignment followed by clustering/classification through Neighbor-Joining (NJ). Initial attempts to cluster the A-domains according to substrate specificity using their whole sequence and the NJ-algorithm were only partly successful [36], [37]. In the case of the complete AT domains, the algorithm enabled the separation of the clusters for the two main substrates; malonyl-CoA (MC) and methylmalonyl-CoA (MMC). However, identification of other substrate clusters appeared far more difficult as they were ‘caught up’ within the two major clusters [17]. Moreover, Yadav et al. [25] reported that five malonyl-specific AT domains (including [PDB:1MLA]) did not cluster with the majority of malonyl-specific AT domains. In another analysis, the malonyl-specific RapC was found within the MMC clade [38]. As mentioned before, residue selections have been made to improve the prediction. For the A domain a selection was made from the so-called core motifs [36], [39], [40], [41] and then the selection was further restricted to the active site residues [26], [27], [28]. Similarly in the case of the AT domain, the selection was at first restricted to the active site and some adjacent residues [24], [25] and later extended [31], [32].

Although the tools perform well in predicting the substrate specificity of the AT and A domains for many substrates, for some substrates they perform less well [31], [32]. These substrates include for instance ethylmalonyl-CoA (EMC) and methoxymalonyl-CoA (MOMC) which are being classified together with malonyl- and methylmalonyl-CoA (MC and MMC) in the case of the AT domains. In addition, the performance of the tools with respect to new sequences depends critically on multiple sequence alignment and the correct extraction of active site residues, which makes the performance very sensitive to the quality of the new alignment. We decided to evaluate the substrate specificity prediction for the AT and A domains of PKSs and NRPSs. Similar to what others have done, we used only AT and A domain sequences related to experimentally validated substrate specificity. We have created Hidden Markov Models (HMMs) to reduce the alignment dependency in case of the allocation of putative substrate specificities to AT and A domains that have not been experimentally characterized. In particular, the use of these HMMs proved to be a crucial step in achieving a high prediction accuracy. This finding corroborates the success of two recent A domain substrate prediction tools NRPSsp [42] and NRPS predictor2 [43]. Moreover, we found that the quality of the prediction could be improved further by using ensembles of HMMs.

## Materials and Methods

### Sequence data

Sequence data from experimentally verified NRPSs and PKSs of bacteria and fungi were taken from the reference databases NRPSDB, PKSDB [29] and ASMPKS [44]. Additional sequence data of experimentally characterized NRPS/PKS systems, as found via literature searches in Pubmed, were taken from NCBI [45] [http://www.ncbi.nlm.nih.gov] and UniProt [46] [http://www.uniprot.org]. The list of sequences and the appropriate literature references are given in sheet 1 of File S1 and File S2. The list contained 213 AT domain sequences and 498 A domain sequences, respectively. In case of the A domains, the dataset that was recently published by [43] and was provided as supplementary ‘original’ and ‘new’ data (546 sequences), was added. The domain sequences obtained from [43] were extended on basis of the protein identifier and the related entries in the UniProt database. To identify and extract the domain boundaries of the A and AT core domains, the NRPS-PKS tool [29], the PKS/NRPS Analysis tool [30] and ASMPKS [44] were used. The combined set of A domain sequences is given in sheet 2 of File S2 (1044 sequences). For testing purposes we downloaded the A domain sequence set provided by [42] [http://www.nrpssp.com] (1546 sequences; given in sheet 1 of File S3). However, this dataset contained many sequences for which the function has been inferred on basis of sequence alone (as can be concluded from the associated information in the Uniprot database [46]), and it contained a considerable number of sequences not related to NRPSs but to enzymes such as D-alanine–poly(phosphoribitol) ligase and Phenylalanine racemase (see sheet 2 of File S3). Besides, we found a few verifiable erroneous annotations in the data-set.

### Multiple sequence alignment

A multiple alignment of the AT domain sequences was made using ClustalX [47] and of the A domain sequences using MAFFT [48] (default settings). The most important feature we used to judge the usefulness of the alignment was the homogeneity (i.e. well aligned and low number of gaps) of conserved parts for all substrate groups, as this feature enhances the comparability of the substrate specific sequence models. To increase the homogeneity of the alignment, the extending residues at the N-terminus and/or C-terminus were removed and the reduced sequences re-aligned. The procedure was repeated until either extensions or gaps were absent from the N-terminus and C-terminus. The reduced and aligned sequences are given in sheet 2 of File S1 and sheet 3 of File S2. From the final alignments Neighbor-Joining (NJ) trees were generated using ClustalX [47]. The NJ trees were visualized using Dendroscope [49] or LOFT [50] and were rooted using the latter program. The multiple sequence alignments and corresponding neighbor joining trees can be found in raw format in the ‘Alignment’ and ‘NJtrees’ directories at http://www.cmbi.ru.nl/bamics/supplementary/Khayattetal_2012_NRPSPKS/.

### Selection of substrate specificity related residues

The residues of the aligned AT and A domain sequences were numbered according to the AT domain of *E coli* FabD [PDB∶1MLA] [24] and the A domain of GrsA [PDB∶1AMU] [26], respectively. Then, the conserved residues (100% identity) were identified within each subset of sequences related to a particular substrate and these were collected as reduced sequences in separate files (see the ‘Alignment’ directory at http://www.cmbi.ru.nl/bamics/supplementary/Khayattetal_2012_NRPSPKS/.). For reasons of comparison, the previously identified sets of characteristic residues according to [24], [25], [28], [35] were also collected in separate reduced sequence files. The following sets of reduced sequences were considered in our analysis: i) sequences composed of all positions (residues) that show absolute conservation for at least one particular substrate, (a) including or (b) excluding all positions that show conservation for all substrates; ii) sequences composed of all positions (residues) that show absolute conservation in at least half of the particular substrates (for the AT-domain). The residues were extracted using Jalview [51]. Sequence conservation was visualized using Weblogo [52] [http://weblogo.berkeley.edu/].

### The creation of substrate specific Hidden Markov Models

It appeared that both the AT and A domain data-sets contained many duplicate or near duplicate sequences. To ensure a balanced coverage of the available sequence space, we removed the (near) duplicate sequences. In this way a non-redundant set of 167 AT domain sequences and 571 A domain sequences remained, as indicated in sheet 2 of File S1 and sheet 3 of File S2. Substrate specific Hidden Markov Models were created using HMMER (version 2.3.2) [53] on basis of the alignment of the non-redundant sets of reduced sequences. In this way 8 substrate specific AT-domain HMMs and 39 substrate specific A-domain HMMs were made. We will refer to these models as the single HMMs. To enhance the predictive value multiple HMMs were generated for those substrates that were well-represented in the datasets (i.e. 2–4 models for those sequences present at numbers ≥10 for AT and ≥15 for A domains). The division was made on basis of the observed grouping in the substrate specific NJ trees. We will refer to the total of these models as an ensemble of HMMs. To estimate the dependency of the various models on the composing sequences, a leave one out cross validation was performed. For every group of sequences a specific HMM was made on basis of all members minus one, and that sequence was then scored with the new model. The procedure was repeated until all sequences had been left out once. The results of the analysis can be found in sheet 3 of File S1 (AT domains) and sheet 5 of File S2 (A domains).

### Implementation of the predictive Hidden Markov Models

The HMMs were implemented in a straightforward manner using Python. The associated web-tool can be found at [http://www.cmbi.ru.nl/NRPS-PKS-substrate-predictor/]. The tool provides the opportunity to paste or upload domain sequences and select the appropriate HMMs to analyze these sequences. To ensure a proper prediction it is essential to use only the sequence of the A or AT domain from the complete NRPS or PKS sequence, respectively. To identify and extract the domain boundaries from the protein sequence of the complete system we advise to use the search domain option in either of these tools NRPS–PKS [29], PKS/NRPS Analysis [30] or ASMPKS [44]. The analysis results are given in html format and contain the substrate annotation related to the best scoring HMM together with the associated e-value and similarity bit score. We observed that in case the bit-score was below 325 (AT domains) or 625 (A domains) the prediction became less reliable and therefore these bit-scores were used as threshold. The HMM profiles for the AT and A substrate groups were compiled in two separate substrate specific HMM libraries that can be found in the ‘HMMs’ directory at http://www.cmbi.ru.nl/bamics/supplementary/Khayattetal_2012_NRPSPKS/. A representation of the presented workflow can be found in Figure S1.

## Results and Discussion

**Figure 1:**
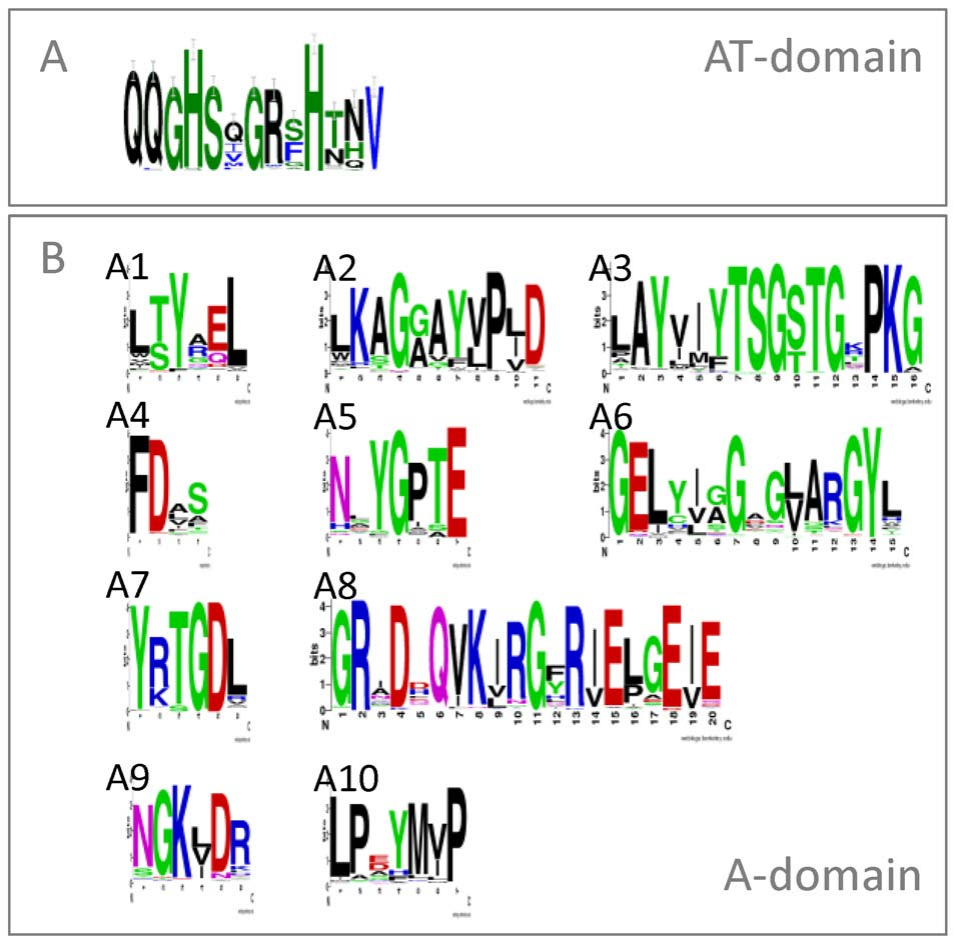
Frequency representations of conserved residues in the AT- and A-domain. A) the active site residues extracted for the AT domain and B) the 10 core motifs within the A domain. The representations were made using Weblogo [52] on basis of the multiple sequence alignment of all domains in the collected dataset and the 13 active site residues identified by [24] (i.e. 11, 63, 90–94, 117, 200, 201, 231, 250, 255) for the AT domain and the 10 core motifs identified by [54] for the A domain.

A comprehensive set of AT and A domain sequences was collected from reference databases and from the literature (see methods, Figure S1, and sheet 1 of File S1 and File S2). After removal of duplicate and near-duplicate sequences, whose presence might bias the analysis, the set included 167 AT and 571 A domain sequences and represented 12 and 58 different substrates, respectively. The published A- and AT-domain substrate prediction methods are mostly based on a selection of catalytic site residues. Therefore, the overall conservation of the catalytic residues of the AT domain, as defined by [24], and of the residues constituting the 10 core motifs (A1–A10) of the A domain, as defined by [54] was determined for the complete set of sequences (results in Figure 1). Many residues appeared completely conserved whereas notable variations between groups of substrates were observed for other residues. Moreover, some of the core residues showed variability within particular groups of substrates. Considering the difference in conservation patterns between the residues, we decided to again evaluate the choice of the residues that are taken into account for the *de novo* prediction of substrate specificity.

## Analysis of the acyl-transferase (AT) domains

The NJ tree that was created on basis of the alignment of all 213 initially collected AT domain sequences showed a clear separation between the sequences related to malonyl-CoA and those related to methylmalonyl-CoA (Figure S2). In contrast, sequences related to methoxymalonyl-CoA and ethylmalonyl-CoA did not end up in distinct clades. This observation is in line with earlier findings [17], [25], [38], [55]. The above ‘separation’ problem observed when using the complete domain sequences has been partly overcome by limiting the number of aligned residues to the active site residues [24], [25], [29], [31], [32], or to the conserved residues. The latter were referred to as quantitative evolutionary traces in the method of [35]. Based on the multiple sequence alignment and residue selections as reported in literature, new NJ trees were generated and the separation of the different substrate specificities into distinct clusters was evaluated. The results are summarized in columns 3, 4 and 5 of Table 1.

**Table 1:** AT domain classification on basis of the NJ-algorithm for various selected sets of residues.

AT Domain Substrate^$^	Complete this study	13 residues Serre et al.	23 residues Yadav et al.	92 residues Minowa et al.	165 or 146^a*^ selected residues	37^b*^ selected residues
MC (92)	1∼	0.96∼	0.95∼	0.98∼	0.90∼	1∼
MMC (83)	1∼	1∼	1∼	1∼	0.96∼	1∼
2MBuC (2)	nsc	1	1	nsc	1	1
IBuC (3)	0.66	0.66	0.66	0.66	0.66	1
PC (3)	1	1	1	1	1	1
MOMC (12)	nsc	nsc	nsc	nsc	nsc	1
EMC (12)	nsc	nsc	nsc	nsc	nsc	nsc

The first column lists the different substrate groups and gives the number of represented sequences between brackets. The values in columns 3, 4 and 5 were calculated on basis of the residues identified by [24], [25] and [35], as indicated. The two major substrate groups MC (malonyl-CoA) and MMC (methylmalonyl-CoA) were reasonably well distinguishable in all trees. However, the factual accuracy of the MC and MMC prediction is lower than 1 as all of the ‘minor’ substrate specific AT sequences fall within the both clusters. Abbreviation: nsc, not in a single cluster.

**Figure 2:**
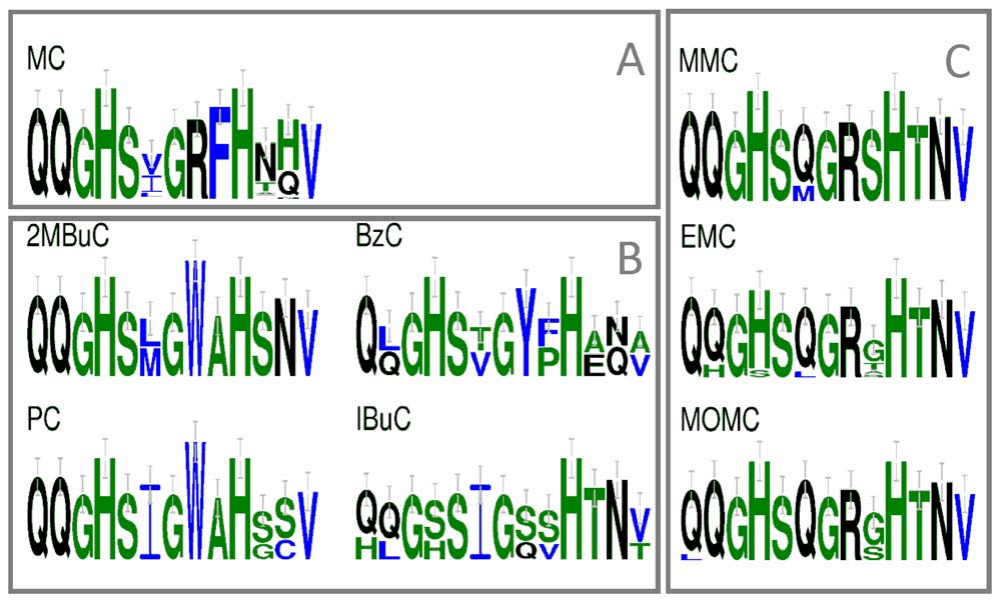
Frequency representation of the active site residues within the AT domain per substrate. The Malonyl CoA (MC) specific AT domain can be separated from the rest on basis of a clearly distinct conserved residues (box A) and likewise can the 2-Methylbuteryl-CoA (2MBuC), the Benzoyl-CoA (BzC), the Isobuteryl-CoA (IBuC) and the Propionyl-CoA (PC) specific AT domains (box B); For the MMC, Methylmalonyl-CoA (MMC), the Ethylmalonyl-CoA (EMC) and the Methoxymalonyl-CoA (MOMC) specific AT domains the conserved active site residues are almost indistinguishable (box C). The sequence representations were made using weblogo [52].

$ For substrate abbreviations see the legend of Figure 2. The initial complete dataset was used to compose the Table (i.e. including the near duplicate sequences), excluding the sequences related to BzC (2), 3MbuC (1), AC (1), CH (1), and CP (1).

a* 165 conserved positions (100% identity) in at least one of the substrate groups; 146 conserved positions in case the residues are removed that are conserved throughout all substrate groups; b* Conserved positions (100% identity) in at least three of the substrate groups (do not include global identical).

Remarkably, the most restricted set of residues, involving only those related to catalysis [24], provided as good a separation as the larger sets used by [35] and [25]. We compared the conservation of the catalytic residues of the AT domain for every individual substrate and found that it would be very hard or even impossible to distinguish between some substrates on basis of these residues alone (see the sequence logo's of MMC, EMC and MOMC in Figure 2). We therefore made two new residue selections by identifying within the complete multiple sequence alignment all those residues that were fully conserved within each group of sequences related to a particular substrate (see methods and Figure S3). Based on this collection of conserved residues, a NJ tree was created and the clustering for the various substrates was inspected (column 6 Table 1). The distinction between the various substrates appeared not better or worse than that observed for the other sets. We succeeded in reducing the statistical noise induced by greater numbers of identical residues in small substrate specific sets of sequences by limiting the selection of residues to those that were conserved in at least 3 (out of 7) substrate specific sets of sequences. By doing so, the resulting NJ tree showed a perfect distinction for 6 of the specific substrates, including MOMC, and this was better than reported before (Table 1 column 7).

## Analysis of the adenylation (A) domains

**Figure 3:**
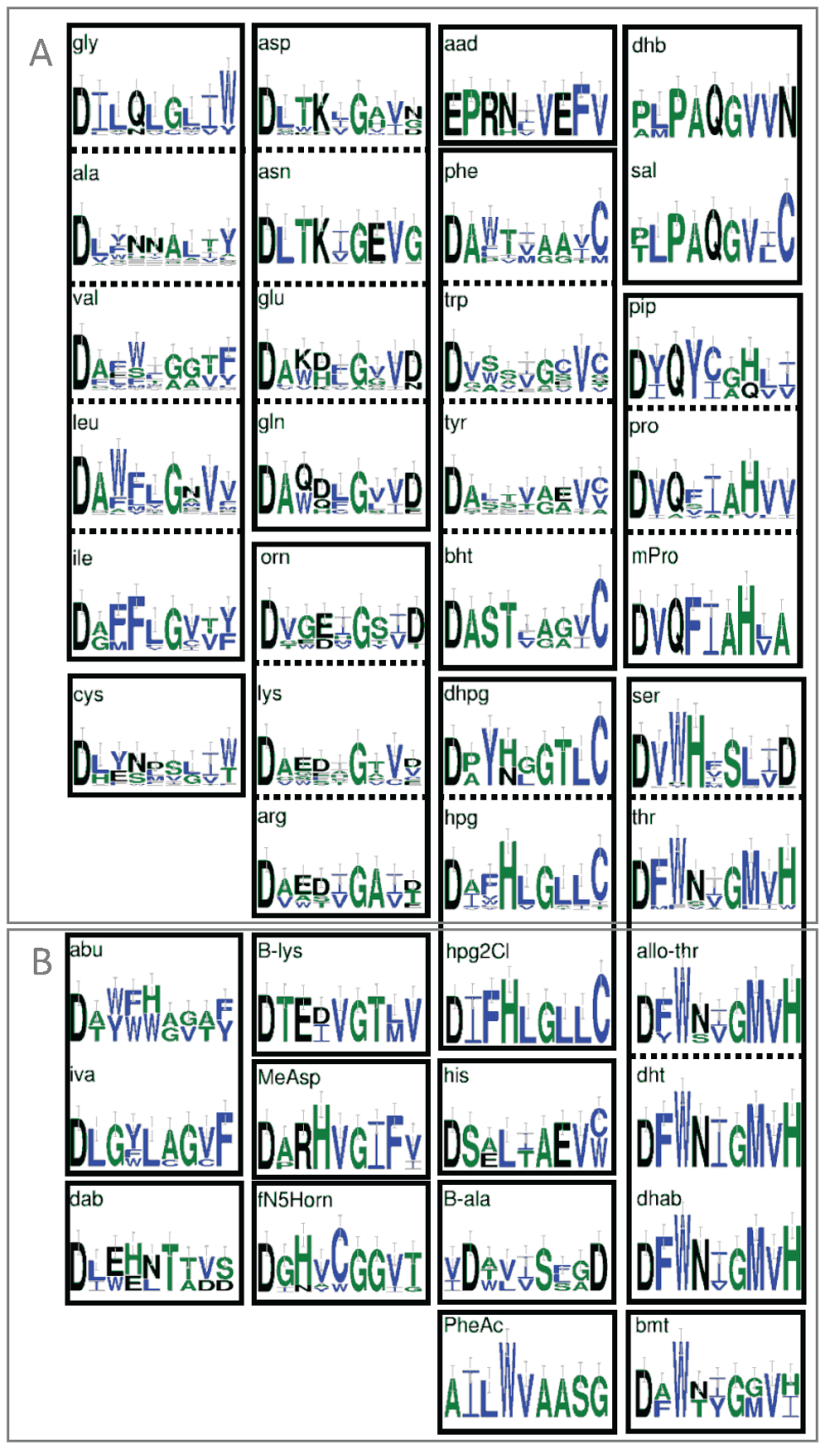
Frequency representation of the active site residues within the A domain per substrate. A) The A-domains were clustered according to common conserved residues as indicated by black boxes) (see e.g. [33]). B) The newly identified substrates have been placed on basis of the motif. For proteinogenic amino acids the three-letter code was used. The non-proteinogenic amino acids are indicated by the following abbreviations: aad, 2-amino-adipic acid; abu, 2-amino-butyric acid; allo-thr, allo-threonine; B-ala, beta-alanine; bht, beta-hydroxy-tyrosine; B-lys, beta-lysine; bmt, (4R)-4[(E)-2-butenyl]-4-methyl-L-threonine; dab, 2,4-diamino-butyric acid; dhab, 2,3-dehydroaminobutyric acid; dhb, 2,3-dihydroxy-benzoic acid; dhpg = dpg, 3,5-dihydroxy-phenyl-glycine; dht, dehydro-threonine = dhbu = 2,3-dehydroaminobutyric acid; fN5horn, N5-hydroxyornithine; hpg, 4-hydoxy-phenyl-glycine; hpg2Cl, 3,5-dichloro-4-hydroxy-L-phenylglycine; iva, isovaline; masp, methyl-aspartate; mpro, methyl-proline; orn, ornithine; pheac, phenylacetate; pip, pipecolic acid; sal, salicylic acid; sar, sarcosine. The sequence representations were made using Weblogo [52].

The NJ algorithm did not suffice to cluster many A domain related substrate groups in separate clades when a multiple sequence alignment of the complete set of domain sequences was used (Figure S4). This observation is in line with the findings of [27]. Figure 3 depicts the residues of the catalytic site, as defined by [26], for the various substrates related to the A domain. Similar to the case of the AT domain, the figure implies that a selection of only the active site residues of the A domain should provide a separation into sub-groups for different substrates, but probably would not be sufficient to predict specificity more precisely. To compare the predictive potential of the residue sets that have been proposed in the literature, these sets were extracted from the complete sequence and a NJ tree was made after their alignment. However, the NJ algorithm failed to create clear clusters for many of the A domain related substrate groups (not shown).

## Rationale for the Creation of Substrate Specific Hidden Markov Models (HMMs)

In the early studies of [27], [28] on the classification of A domains, the low number of available A domains of experimentally determined substrate specificity obscured the inherent imperfectness of classification by NJ. The imperfect classification predominantly seems to arise from the fact that the precise positioning of residues within a multiple sequence alignment, which is extremely important in case conserved residues are to be selected, appeared very sensitive to the variability within the set of aligned sequences. To circumvent the positioning problem of single sequences we decided to create substrate specific Hidden Markov Models, which are far less sensitive to this phenomenon. Although initially a multiple sequence alignment is used to create them, the classification of a new query sequence does not require *a priori* alignment. Substrate specific HMMs were created after a multiple-sequence alignment of the complete set of sequences within the non-redundant dataset on basis of the aligned sequences related to a particular substrate (see methods). We used the complete alignment to create the HMMs to reduce potential noise caused by small sample sizes and to increase the comparability of the HMM scores. Then, as a first test, each sequence within the non-redundant AT and A domain datasets was scored with all eight profiles in the AT- and thirty nine profiles in the A- substrate specific HMM libraries, respectively. The scores were compared and it appeared that in most cases the correct HMM provided the highest score.

## Substrate specific HMM-based classification of AT Domains

The results of the HMM analysis of the AT domain sequences are given in sheets 3 and 4 of File S1 and are summarized in Table 2. The HMMs that were generated on basis of the aligned reduced domain sequences were tested against the complete domain sequences. A comparison of the results given in Table 1 and Table 2 makes clear that in the case of the AT domains the HMM-based approach improved the predictive power significantly with respect to that reported for the NJ-based approach.

**Table 2:** AT domain classification on basis of HMMs.

	single HMMs			ensemble of HMMs			ensemble of HMMs LOO		
**Substrate^$^**	**c**	f	*at*	**c**	f	*at*	**c**	f	*at*
MC (69)	**65**	1	*3*	**69**	0	*0*	**60**	3	*6*
MMC (63)	**63**	0	*0*	**63**	0	*0*	**62**	1	*0*
2MBuC (2)	**2**	0	*0*	**2**	0	*0*	**0**	2	*0*
BzC (2)	**2**	0	*0*	**2**	0	*0*	**0**	1	*1*
IBuC (3)	**3**	0	*0*	**3**	0	*0*	**2**	1	*0*
EMC (11)	**9**	2	*0*	**11**	0	*0*	**4**	7	*0*
MOMC (10)	**8**	0	*2*	**9**	1	*0*	**6**	4	*0*
PC (3)	**3**	0	*0*	**3**	0	*0*	**3**	0	*0*
Other (4)^#^	0	3	***1***	0	3	***1***			
*Correct (%)*	**92.8**	3.6	*3.6*	**97.0**	2.4	*0.6*	**84.0**	11.7	*4.3*

The first column lists the different substrates and between brackets the number sequences that were analyzed. The Table lists the number of correctly (c, bold) and falsely (f) classified sequences and the number of sequences that scored above threshold (at, grey and in italics). The values in columns 2, 3 and 4 were derived from the use of a single HMM per substrate, and the columns 5, 6 and 7 relate to the prediction made using an ensemble of multiple HMMs per substrate. The values in columns 8, 9 and 10 relate to the Leave One Out cross validation.

$ The set contained 167 non redundant sequences. See the legend of Figure 2 for the systematic name of the various substrates.

# The category ‘other’ sequences includes those specific for 3MbuC, AC, CH and CP as only one sequence has been experimentally identified and thus no reliable model could be made.

When using HMMs for the classification of AT domains, the data indicate that it is not necessary to make a selection of residues to achieve an overall high accuracy (i.e. 93% is predicted correctly; see columns 2-4 in Table 2). In fact, three out of the six false predictions involved a substrate that lacked a prediction model and could thus not be appropriately predicted with any of the schemes. We observed a clear variability in HMM scores for various sequences with the same substrate specificity (sheet 3 File S1). This implied that the models included another strong sequence signature besides that related to the specific substrate. Given the nature of sequence evolution it is clear that the additional signature should include the residues that signify the evolutionary kinship. In fact, when we subdivided the sequences related to substrates EMC, MC, MMC and MOMC, in 2-4 groups on basis of their evolutionary relationship as derived from the grouping in the substrate specific NJ trees, and then made HMMs accordingly (i.e. we made an ensemble of HMMs for those substrates), we found that the prediction performance further increased (columns 5-7 in Table 2). To test the sensitivity of the individual models in the ensemble towards the constituent sequences we performed a Leave One Out cross validation (columns 8-10 in Table 2). For the main substrate groups MC and MMC the cross validation makes clear that the related sequence models cover the known sequence variability well. Remarkably, the models for the minor substrate groups, which were based on fewer sequences, proved either sensitive (i.e. 2MBuC, BzC, EMC, iBuC and MOMC) or not at all (PC). At the same time, we observed that the sequences in the latter set were far less divergent. This actually explains the good cross-validation performance. The cross validation thus indicates that the models related to the minor substrates can certainly be improved in case more and diverse sequences are added. Yet our analysis also implies that at present the given ensemble of HMMs almost perfectly represents the currently validated AT domain sequence space.

## Substrate specific HMM-based classification of A Domains

The results of the HMM analysis of the A domains are given in sheet 4 of File S2 and are summarized in Tables 3 and 4. We found that the use of whole domain sequence based HMMs provided an accuracy of prediction that was better than achieved when using a limited set of selected residues as reported by [28], [35] and [31] (not shown). The inability to discriminate between certain substrates based on the supposed active site residues is reflected in the similarity of some of the sequence logos for the extracted active site residues that line the substrate binding pocket of the A domain (see Figure 3). Recently, two other groups have used HMMs and Support Vector Machines ([42] and [43], respectively) to diminish the alignment dependence and improve the prediction quality of the substrate specificity for A domains. We compared the performance of our single HMMs with the performance of the related tools NRPSsp [42] and NRPS predictor 2 [43] using those sequences that were used to construct the other predictors and that were shared with our dataset (as indicated in sheet 4 of File S2). In fact, our dataset includes all the data used to train the latter tool. We found that the recovery of correct links between sequence and substrate was somewhat higher using the single HMMs than using the published tools (see Table 3).

**Table 3:** Quality of A domain substrate specificity predictions using HMMs and SVMs.

	data^$^	correct	false	Above threshold	coverage	Correct of covered
NRPSsp	P∩K’	**86**	7	*7*		
	K				(77)	**90**
NRPSpredictor2	R∩K’	**85**	8	*7*		
	K				(79)	**90**
single HMMs	K’	**93**	4	*3*		
	K				(88)	**95**
ensemble HMMs	P∩K’	**99**	1	*0.3*		
	R∩K’	**96**	3	*0.7*		
	K’	**97**	2	*0.6*		
	K	**92**	4	*4*	(96)	**96**
	P	**85**	3	*12*	(88)	**96**
	LOO	**66**	13	*21*	(79)	**84**

Substrate specificity predictions were made for various sequence data-sets using the published tools NRPSsp [42], NRPSpredictor 2 [43], and our single and ensemble of HMMs. Column 1 indicates the predictor that was tested and Column 2 the data that was used to test. Columns 3 and 4 provide the percentage of correct and false predictions below the set threshold, respectively, and column 5 the percentage of predictions that scored above threshold. Column 6 gives the fraction of sequences from the complete non-redundant data-set that received an annotation. Column 7 provides the fraction of correctly annotated sequences within the set of sequences that was provided with an annotation.

$ To test the coverage and check the validity of the predictions, the four predictors were applied to the non-redundant reference dataset of experimentally validated substrate specific A domain sequences collected by us from the reference databases, literature and from [43] (set K = 571 sequences). To compare the performance, the predictors were applied to those sequences that are shared between data-sets. We found 392 sequences to be shared between the data-set used to train NRPSsp [42] and our non-redundant set (P∩K’), and 405 sequences to be shared between the data-set used to train NRPSpredictor2 [43] and our non-redundant set (R∩K’). In this case, K’ indicates that the sequences related to a substrate for which no model was present in either of the predictors, were left out in the comparison. The ensemble of HMMs was also applied to the dataset provided by [42] (P). To test the sensitivity of the ensemble models with respect to the removal of constituent sequences a Leave One Out cross validation was performed (LOO).

**Table 4:** A domain classification with an ensemble of HMMs.

	ensemble HMMs			LOO		
**Substrate^$^**	**c**	f	*at*	**c**	f	*at*
aad (10)	**10**	0	*0*	**9**	0	*1*
abu, iva (17/12)*	**15**	1	*1*	**8**	3	*1*
ala (46)	**45**	1	*0*	**26**	8	*12*
b-ala(4)^ #^	**4**	0	*0*	**0**	0	*4*
arg (7)	**7**	0	*0*	**2**	1	*4*
asn (20)	**20**	0	*0*	**13**	0	*7*
asp (15)	**15**	0	*0*	**9**	0	*6*
bht (6)	**6**	0	*0*	**5**	1	*0*
bmt (2) * ^#^	**2**	0	*0*	**0**	2	*0*
cys (27)	**26**	0	*1*	**19**	2	*6*
dab (10)^ #^	**10**	0	*0*	**9**	0	*1*
dhab, dht (4)^ #^	**4**	0	*0*	**4**	0	*0*
dhb, sal (12)	**12**	0	*0*	**12**	0	*0*
dhpg, dpg (8)	**8**	0	*0*	**8**	0	*0*
fN5H-orn (4) * ^#^	**4**	0	*0*	**4**	0	*0*
gln (10)	**10**	0	*0*	**6**	3	*1*
glu (16)	**16**	0	*0*	**12**	3	*1*
gly (30)	**29**	1	*0*	**20**	5	*5*
his (2) * ^#^	**2**	0	*0*	**0**	0	*2*
horn (3)^ #^	**3**	0	*0*	**1**	1	*1*
hpg, hpg2Cl (21/15)	**21**	0	*0*	**12**	0	*3*
hyv-d (3)^ #^	**3**	0	*0*	**0**	0	*3*
ile (13)	**13**	0	*0*	**10**	3	*0*
leu (41)	**37**	4	*0*	**31**	7	*3*
lys (8)	**8**	0	*0*	**0**	0	*8*
b-lys (3) * ^#^	**3**	0	*0*	**2**	0	*1*
me-asp (4) * ^#^	**4**	0	*0*	**4**	0	*0*
orn (12)	**12**	0	*0*	**6**	1	*5*
phe (15)	**14**	1	*0*	**4**	5	*6*
phe-ac (3) * ^#^	**3**	0	*0*	**3**	0	*0*
pip (8)	**8**	0	*0*	**3**	2	*3*
pro, me-pro (20)	**20**	0	*0*	**14**	1	*5*
ser (33)	**29**	3	*1*	**25**	4	*4*
thr, allo-thr (34)	**34**	0	*0*	**30**	2	*2*
trp (14)	**14**	0	*0*	**5**	2	*7*
tyr (18)	**18**	0	*0*	**9**	6	*3*
val (34)	**33**	1	*0*	**24**	4	*6*
ambiguous (15)	**5**	4	*6*	**-**	-	*-*
other (19) * ^#&^	0	4	***15***	**-**	-	*-*

The first column lists the different substrates and the number of sequences analyzed (between brackets). The second column lists the number of correctly classified sequences by our ensemble of HMMs, for the non-redundant reference dataset of experimentally validated substrate specific A domain sequences collected from reference databases, literature and from [43] (set K = 571 sequences). The third column gives the number of sequences that received a false annotation (f), and the fourth column gives the number of sequences that scored above treshold (at, grey and numbers in italics). Columns five, six and seven provide the same information but then related to the Leave One Out cross validation.

$ See the legend of Figure 3 for the systematic name of the various substrates. The category ‘other’ includes those substrates that are represented only once in the domain sequence dataset. They include: 2-oxo-isovaleric-acid, 3-methyl-glutamate (3-me-glu), 4-propyl-proline (4ppro), 2-amino-9,10-epoxy-8-oxodecanoic acid (aeo), alaninol, alle, alpha-hydroxy-isocaproic acid, an, (S)-2-amino-8-oxodecanoic acid (aoda), l-capreomycidine (cap), d-lysergic acid (d-lyserg), hydroxyl-asn, hmp-D, LDAP, MeHOval, N-methyl-phenylalanine (mephe), N-methyl valine (meval), N-(1,1-dimethyl-1-allyl)tryptophan, phenyl-glycine (phg), s-nmethoxy-tryptophan, (4S)-5,5,5-trichloro-leucine (tcl), valinol (vol).

* , # and &: For particular substrates no representative models were present in one or more of the predictors that were compared in Table 3 (*, NRPSsp; #, NRPS predictor 2; &, ensemble HMMs). Ideally the related sequences should obtain a score above threshold.

We also observed differences between the three predictors for both the number of recognized (i.e. covered) sequences and the number of correctly assigned in the case of various substrates. We attribute this phenomenon to the way substrate specificity evolves in NRPSs and PKSs, and the fact that the predictors have been trained on different sets of sequences. In case the NJ trees that were constructed on basis of the alignments for the AT as well as for the A domains (Figure S2 and Figure S4) are taken as representative for the sequence evolution of NRPSs and PKSs, the occurrence of the same substrate specificity in different clades of the tree should be interpreted as the consequence of a diversification of function between closely related homologous domains or even orthologous domains so that they acquired the same function as more distantly related homologous domains (i.e. the formation of analogs within a set of homologs). Such an evolutionary path inevitably has a negative effect on the predictive power of single sequence models in case the residues that were conserved due to evolutionary kinship outnumber the residues that have been conserved due to identical substrate specificity. The difference between these numbers will be especially large in case only a relatively small number of sequences from particular evolutionary branches are used to build the sequence models. For instance, we have based the substrate specific sequence models in all cases on a limited set of sequences (∼5–50). As a consequence, our models should perform well (i.e. yield high HMM scores) for evolutionary related sequences and perform less well for sequences that followed another evolutionary route towards the same substrate specificity.

Therefore, we also made multiple HMMs to represent single A domain related substrates, like we did earlier for the AT domains. Again we found that the ensemble of HMMs clearly out-performed the single HMMs (i.e. combining a higher coverage and a higher accuracy). In fact, it is well known that ensemble methods can be used for improving prediction performance, provided that the classifiers are independent [56]. We tested the ensemble on the dataset of 1546 A domain sequences collected by [42] and found that the percentage of covered sequences dropped slightly from 96% to 88%, which might indicate that the coverage of the sequence space by the ensemble HMMs could be improved by addition of more sequences (see Table 3). The performance was better than the reported performance of NRPSsp, which was actually trained on this dataset. Nevertheless, the numbers should be interpreted with some care as the dataset contained many sequences for which the link between substrate and experimental evidence is not traceable. In addition, the set contained a considerable number of sequences not related to NRPSs but to enzymes such as D-alanine–poly(phosphoribitol) ligase and Phenylalanine racemase. In fact a substantial number of the sequences that scored above threshold, and thus reduced the coverage, related to the alanine-ligase (see sheet 2 of File S3).

The performance of the ensemble predictor appeared substrate dependent (data in Table 4). The published tools performed less well, but predominantly on only a limited number of substrates (results listed in sheets 4, 6, 7 and 8 of File S2). For instance, the predictor NRPSsp performed poorly for ala, glu and phe. This is probably caused by the fact that their training data for ala and phe contained many enzyme sequences not related to NRPSs and the glu-related sequences contained a few erroneous annotations (see sheet 2 of File S3). In the case of NRPS predictor 2, the predictor lacks a number of sequence models like that related to 2,3-dehydroaminobutyric acid (dhab/dht) and 2,4-diamino-butyric acid (dab).

We performed a Leave One Out cross validation to establish the sensitivity of the various models towards the constituent sequences (results in sheet 5 of File S2 and summarized in Tables 3 and 4). We found that the overall performance clearly dropped when removing a sequence from each model (Table 3). This is indicative of an imperfect coverage of the total sequence space by the as yet experimentally validated A domain sequences. We observed that some substrate models proved rather sensitive to the constituent sequences whereas others were not, very similar to what we found with the AT domains (sheet 5 in File S2). In most cases this difference reflects the divergence in the sequences that constitute the model. Given the proposed evolutionary path of the A domain sequences, this kind of sensitivity is actually inevitable. In various cases there is only a single representative sequence with a particular substrate specificity among several evolutionary closely related sequences with a different substrate specificity. The cross validation makes clear that several models can certainly be improved in case more and diverse sequences could be added. Yet our analyses at the same time imply that the given ensemble of HMMs best represents the currently validated A domain sequence space.

## Implementation

To enable substrate predictions based on the HMMs that we have used, a simple web tool was implemented. The tool allows a user to paste or upload a single sequence or a set of sequences and then to run a particular set of HMMs. The tool requires the domain sequence as input, which can be obtained by using the search domain option in either of these tools NRPS–PKS [29], PKS/NRPS Analysis [30] or and ASMPKS [44]. The ensemble of HMMs is used to generate a substrate prediction based on the best scoring model. The implementation and appropriate use is described in the methods section. The tool can be found at [http://www.cmbi.ru.nl/NRPS-PKS-substrate-predictor/].

## Conclusions

It has been argued that the accuracy of the substrate specificity prediction tools for the A and AT domains of NRPS and PKS systems was mainly limited by the relatively low numbers of experimentally characterized A and AT domains [32]. Our current work shows that this is only partially true. Previous classification efforts were based on the extraction of particular active site residues [25], [27], [28], [33] and thereby rested on the assumption that the A and AT domains are all adopting folds and active site geometry similar to those of the structural models [PDB∶1AMU] and [PDB∶1MLA]. However, this is not necessarily the case [57]. Therefore, it is not per definition straightforward to identify the correct active site residues from a multiple sequence alignment only.

The set of sequences that we collected allowed for the creation of substrate specific HMMs that could resolve the specificity for known sets of A and AT domains with higher accuracy. Moreover, the prediction procedure does not depend on the correct alignment of the new sequence and selection of particular residues. The accuracy is mainly limited by the fact that for several substrates the HMMs are biased as a result of the limited set of substrate specific input sequences that could be used to create them. It is therefore to be expected that the power of the approach will increase when more experimentally characterized sequences can be incorporated into the models. In addition, we argue that singular HMMs are not sufficient due to the nature of the evolutionary path towards substrate specificity and the presence of homologous analogs. We show that the ensuing classification problem can be solved by using ensembles of HMMs for the same substrate. These ensembles can be optimized when the constituent HMMs are made evolutionary path specific

The ability to identify substrate specificity of the A and AT domains will not only aid the identification of the final bioactive peptides and polyketides produced by the NRPSs and PKSs, but can also help to rationalize product engineering within the cell by implication of those residues that affect the specificity and those metabolites whose concentration will affect product formation.

## Supporting Information

Figure S1 **Representation of the classification workflow.**
(TIF)

Figure S2 **Neighbor Joining tree of the acyl-transferase domains.**
(TIF)

Figure S3 **Illustration of the criteria that were applied for residue selection in the AT domain.**
(TIF)

Figure S4 **Neighbor Joining tree of the adenylation domains.**
(TIF)

File S1 **Substrate prediction of AT domains and related data.** In sheet 1 the annotated AT domain sequences and related PMID references are given. In sheet 2 the set of reduced and aligned AT domain sequences are given. The final columns indicate whether the sequences were included in creating the substrate specific HMMs. In sheet 3 the results of the HMM and LOO analysis are given. Sheet 4 summarizes the analysis results.
(XLSX)

File S2 **Substrate prediction of A domains and related data.** In sheet 1 the annotated A domain sequences and related PMID references are given. In sheet 2 the combined set of validated A domain sequences is given. Duplicate and near-duplicate sequences were identified and marked. In sheet 3 the non-redundant set of reduced and aligned AT domain sequences are given. The final column indicates whether the sequences were included in creating the substrate specific HMMs. In sheet 4 the results of the HMM analysis are given and in sheet 5 the results of the LOO cross validation. Sheet 6 summarizes the analysis results for the non redundant data-set. Sheets 7 and 8 summarize the analysis results for the non-redundant data that were used to construct the predictors and that were shared.
(XLSX)

File S3 **Substrate prediction of A domains for dataset taken from Uniprot.** In sheet 1 the annotated sequence data as provided by [42] [http://www.nrpssp.com] are given. In sheet 2 the results of the HMM analysis are given. The annotation data related to supposedly wrong predictions and predictions below threshold were looked up and evaluated. Sheet 3 summarizes the analysis results. Sheet 4 provides an overview of the sequences that were present in both the non redundant data-set as well as the data-set from [42].
(XLSX)
