# Supplementary figures and images for: Classification of the Adenylation and Acyl-Transferase Activity of NRPS and PKS Systems Using Ensembles of Substrate Specific Hidden Markov Models
Source: PLoS One. 2013 Apr 18;8(4):e62136. doi: 10.1371/journal.pone.0062136 (PMC3630128; doi:10.1371/journal.pone.0062136)

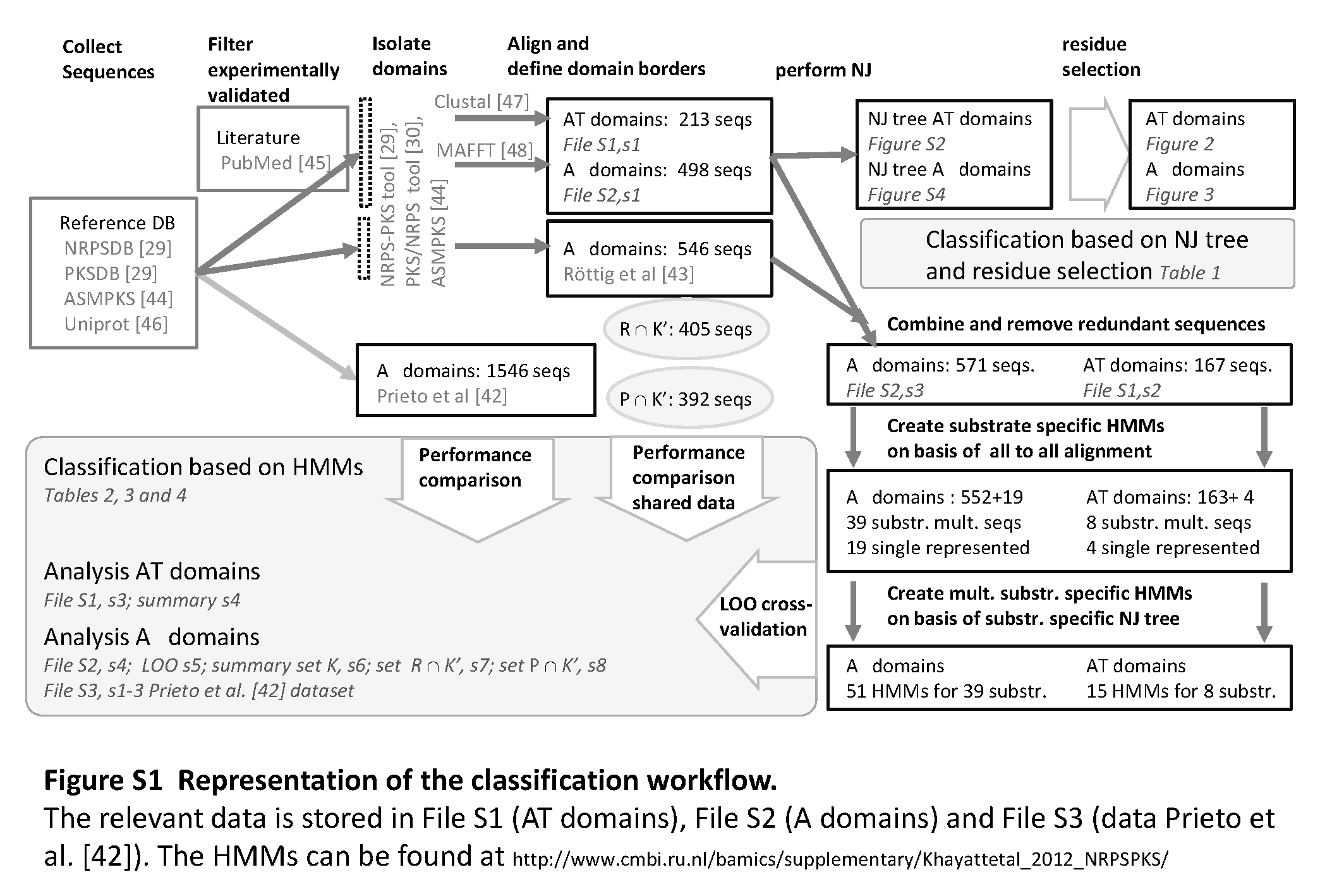

Supplement: Figure S1 — Representation of the classification workflow. (TIF) [file pone.0062136.s001.tif]

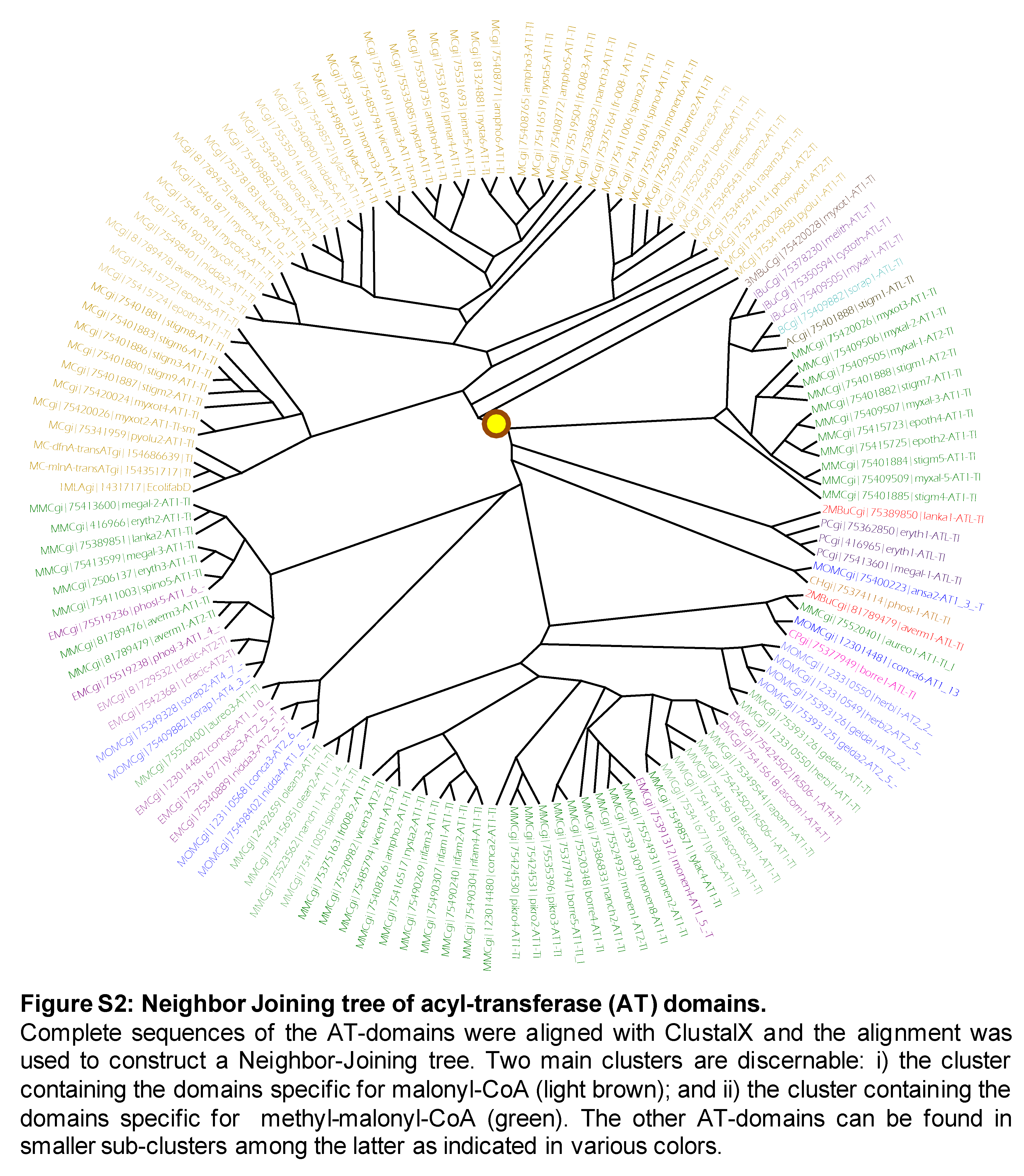

Supplement: Figure S2 — Neighbor Joining tree of the acyl-transferase domains. (TIF) [file pone.0062136.s002.tif]

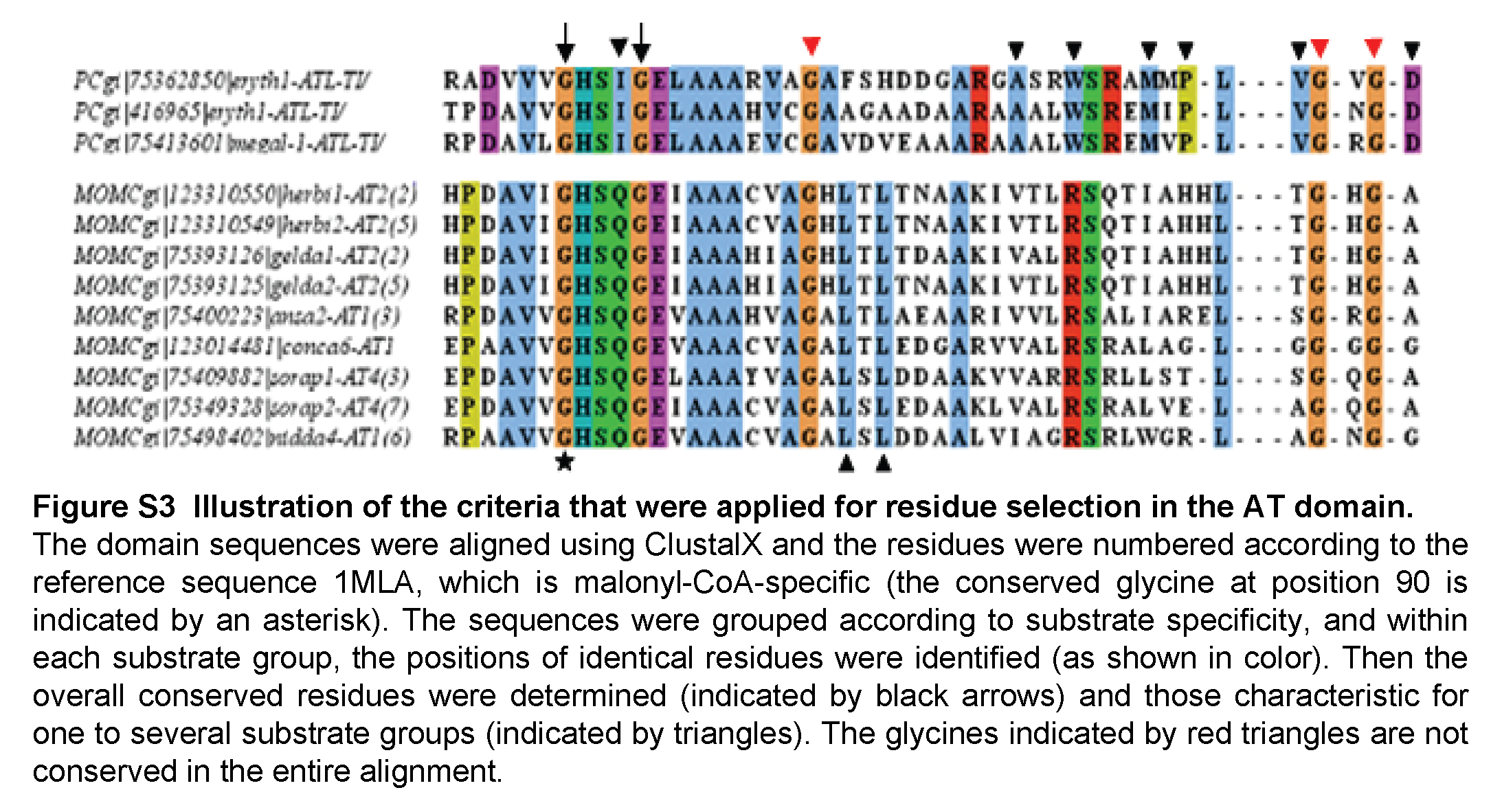

Supplement: Figure S3 — Illustration of the criteria that were applied for residue selection in the AT domain. (TIF) [file pone.0062136.s003.tif]
